# A phase I, single‐center, open‐label study to investigate the absorption, distribution, metabolism and excretion of encorafenib following a single oral dose of 100 mg [ ^14^C] encorafenib in healthy male subjects

**DOI:** 10.1002/prp2.1140

**Published:** 2023-09-29

**Authors:** Lance Wollenberg, Erik Hahn, Jason Williams, Kevin Litwiler

**Affiliations:** ^1^ Pfizer Inc. Worldwide Research Development and Medical Boulder Colorado USA; ^2^ Pfizer Inc. Worldwide Research Development and Medical La Jolla California USA; ^3^ Present address: OnKure Therapeutics Clinical Pharmacology and DMPK Boulder Colorado USA

**Keywords:** BRAF, cetuximab, colorectal cancer, Encorafenib, metastatic melanoma

## Abstract

Encorafenib is a novel kinase inhibitor of *BRAF* V600E as well as wild‐type BRAF and CRAF and has received approval, in combination with binimetinib, to treat *BRAF* V600E or V600K mutation‐positive unresectable or metastatic melanoma or in combination with cetuximab to treat *BRAF* V600E mutation‐positive colorectal cancer. The absorption, distribution, metabolism and excretion (ADME) of encorafenib was studied by administering [^14^C] encorafenib (100 mg containing 90 μCi of radiolabeled material) to 4 healthy male subjects (NCT01436656). Following a single oral 100‐mg dose of [^14^C] encorafenib to healthy male subjects, the overall recovery of radioactivity in the excreta was ≥93.9% in all four subjects, indicating that good mass balance was achieved. An equal mean of 47.2% for the radioactivity dose was eliminated in the feces and urine. The percentage of the dose eliminated in the feces (5.0%) and urine (1.8%) as unchanged encorafenib was minor. Metabolism was found to be the major clearance pathway (~88% of the recovered radioactive dose) for encorafenib in humans and is predominantly mediated through N‐dealkylation of the isopropyl carbamic acid methyl ester to form the primary phase 1 direct metabolite M42.5 (LHY746). Oral absorption was estimated from the radioactive dose recovered in the urine (47.2%) and the total radioactive dose recovered in the feces as metabolites (39%). Based on these values and the assumptions that encorafenib and its metabolites are stable in feces, the fraction of oral absorption was estimated to be at least ~86%.

## Graphical abstract

This phase I study of the absorption, distribution, metabolism and excretion of encorafenib in healthy male subjects found encorafenib was rapidly absorbed and had a reasonable elimination time to support once daily dosing. Encorafenib was primarily eliminated via metabolism of the parent compound, suggesting a key role in hepatic elimination.

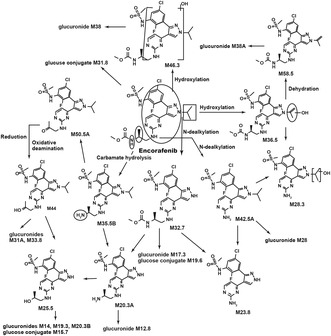

**Abbreviations**
%RA: percent of radioactivity
^14^CO_2_
: radio‐carbon dioxide
ACN: acetonitrile
ADME: absorption, distribution, metabolism and excretion
AE: adverse event
amu: atomic mass unit
AUC: area under the curve
AUC_0‐24_
: area under the curve from time zero to 24 hours post dose
AUC_inf_
: area under of the curve extrapolated to infinity
AUC_last_
: area under of the curve to the last measurable time point
BMI: body mass index
BRAF: B‐Raf proto‐oncogene serine/threonine kinase
CL/*F*
: apparent oral clearance
CLr: renal clearance
*C*_max_
: maximum concentration
CRAF: RAF‐1 proto‐oncogene, serine/threonine kinase
CV%: percent coefficient of variation
DMPK: Drug Metabolism and Pharmacokinetics
DMSO: dimethyl sulfoxide
ECG: electrocardiograms
Fa: fraction of oral absorption
HPLC: high‐performance liquid chromatography
ICH: International Conference on Harmonization
IEC: Independent Ethics Committee
IRB: Institutional Review Board
K2‐EDTA: potassium ethylene diamine tetra acetic acid
LC: liquid chromatography
LLOQ: lower limit of quantitation
LSC: liquid scintillation counting
m/ΔmFWHM: mass resolution
mL: milliliters
mM: millimolar
MS: mass spectrometers
NA: not applicable
ngEq: nanogram equivalent
nm: nanometer
NOAEL: no observed adverse effect level
PFP: pentafluorophenyl
pH: power of hydrogen
PK: pharmacokinetic
QD: once daily
REB: Research Ethics Board
SGF: simulated gastric fluid
SIF: simulated intestinal fluid
*T*_last_
: time to last measurable concentration
*T*_max_
: time to maximum concentration
UPLC: ultra‐performance liquid chromatography
μCi: microcurie
μL: microliters
Vz/*F*
: apparent volume of distribution

## 1 INTRODUCTION

Encorafenib (BRAFTOVI®) is a small‐molecule inhibitor of mutant *BRAF* V600E kinase, with additional cell‐based potency noted against wild‐type BRAF and CRAF. Mutations in the kinase domain of BRAF, specifically found at the V600 site, are frequently found to be drivers of cancer, including a prevalence of mutations noted in, but not exclusive to, melanoma, colorectal cancer, non‐small cell lung cancer and differentiated thyroid cancer.^1^, ^2^, ^3^, ^4^, ^5^ In recent years, progress has been made in improving patient outcomes and extending the survival of patients with *BRAF*‐mutant cancers relative to standard‐of‐care chemotherapeutic approaches using a targeted approach towards inhibition of the kinase activity of *BRAF* V600 mutants.^6^, ^7^ Encorafenib is approved in the United States and elsewhere in combination with binimetinib to treat *BRAF* V600E or V600K mutation‐positive unresectable or metastatic melanoma or in combination with cetuximab to treat *BRAF* V600E mutation‐positive colorectal cancer.

Previously, the pharmacokinetics (PK) of encorafenib as a monotherapy and in combination with binimetinib has been extensively described using traditional bioanalytical methods incorporating mass spectrometric detection.^8^, ^9^ These methods have limited ability to elucidate the metabolic and excretory fate of encorafenib, which is essential to characterize the drug–drug interaction potential of the parent drug and safety of circulating metabolites (>10% exposure related to total administered drug‐related radioactivity). Moreover, these traditional bioanalytical methods provide limited information on the routes of excretion, which is necessary to help identify special patient populations that may experience higher than anticipated drug exposures (e.g., hepatic and renal impairment). To remedy this, a complete characterization of the absorption, distribution, metabolism and excretion (ADME) is described here, by employing a clinical study administering radiolabeled [^14^C] encorafenib drug substance in combination with cold drug substance (~100 mg containing 90 microcuries [μCi] of radiolabeled material).

The primary objectives of this study (ClinicalTrials.gov ID, NCT01436656) were to determine the rates and routes of excretion of encorafenib‐related radioactivity, including mass balance of total drug‐related radioactivity in urine and feces, following the administration of a single 100‐mg dose (90 μCi) of [^14^C] encorafenib in healthy subjects. The safety and tolerability of a single 100‐mg dose (90 μCi) of [^14^C] encorafenib were also evaluated. Additionally, the PKs of encorafenib from direct measurement of the unlabeled drug in plasma and the PKs of encorafenib and its metabolites in blood and plasma as total radioactivity were assessed. Profiling of metabolites in excreta (urine and feces) to determine key biotransformation routes and clearance mechanisms was also conducted.

## 2 MATERIALS AND METHODS

### 2.1 Clinical study design

**FIGURE 1:**
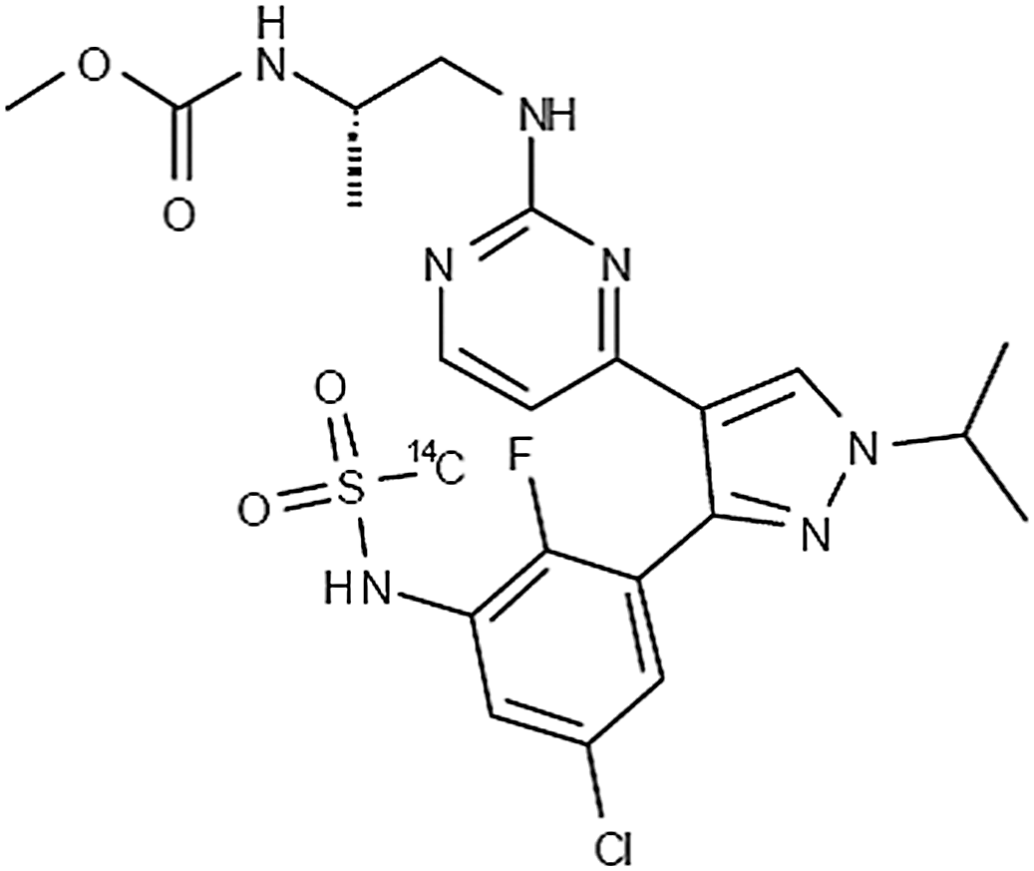
Chemical structure of encorafenib with label placement.

This study (ClinicalTrials.gov ID, NCT01436656) was a single‐center, open‐label study to investigate the ADME of encorafenib after a single oral administration of 100 mg (90 μCi) [^14^C] encorafenib in healthy male subjects. The structure and location of the radioactive label of encorafenib are shown in Figure 1. A total of four evaluable subjects were enrolled and dosed. The study consisted of a screening period, a baseline period, a single dose treatment and a 96 h post‐dose observation period. A safety follow‐up assessment by phone was conducted 30 days after administration of 100 mg (90 μCi) [^14^C] encorafenib. Subjects completed an overnight fast of at least 10 h prior to administration of the study drug. Subjects were discharged from the clinical site provided that the subject's recovery of [^14^C] encorafenib‐related radioactivity was at least 90% of the administered dose. The study duration period could be prolonged if the subject's recovery of related radioactivity was less than 90% by the fifth‐day post‐treatment or the subject had two consecutive 24‐h combined urine and fecal collections yielding ≥1% of dose.

This clinical study was designed, implemented, and reported in accordance with the International Conference on Harmonization (ICH) Harmonized Tripartite Guidelines for Good Clinical Practice, with applicable local regulations (including European Directive 2001/20/EC and US Code of Federal Regulations Title 21), and with the ethical principles laid down in the Declaration of Helsinki. The protocol and the proposed informed consent form were reviewed and approved by a properly constituted Institutional Review Board/Independent Ethics Committee/Research Ethics Board (IRB/IEC/REB) before the study started.

### 2.2 Dosimetry

The planned oral dose of 100 mg of encorafenib contained 90 μCi (3.33 MBq) of [^14^C] per subject. The expected radiation exposure of a subject had been estimated prognostically according to the guidelines of the International Commission on Radiological Protection. The estimate was based on available human PK data, rat ADME data, and rat tissue distribution data using [^14^C]encorafenib (data on file).

The human organ doses were estimated, on the basis of conservative assumptions, by extrapolation of systemic exposure from animals (rat quantitative whole‐body autoradiography and oral rat PK study) to human (based on a human PK of a 100‐mg dose). The whole body committed effective dose was estimated, on a basis of conservative assumptions, to be 0.92 mSv (92 mrem), but the actual effective dose might be lower. The expected effective dose will be below the recommended dose limit for the public of 1 mSv per year and is in compliance with the International Commission on Radiological Protection (ICRP).

### 2.3 Dose preparation

Radiolabeled [^14^C]LGX818 and other ingredients were supplied for the preparation of a cremophor‐based microemulsion. The parent batch of radiolabeled drug substance was prepared by the Novartis Isotope Laboratory (Novartis Institutes for Biomedical Research – Drug Metabolism and Pharmacokinetics [NIBR‐DMPK], East Hanover, NJ, USA). The batch was adjusted for final specific radioactivity by dilution with a non‐radiolabeled drug substance, using a non‐radiolabeled encorafenib released for human use. Each subject, therefore, received a single, oral dose of 100 mg [^14^C]LGX818 formulated as a micro‐emulsion (total dose 3.33 MBq, 90 μCi) and 3.33 mL (or the equivalent calculated weight) of micro‐emulsion was administered to subjects following a 10 h fast.

### 2.4 Collection of biological samples

Venous blood samples (18 mL/time point) were collected by either direct venipuncture or from an indwelling cannula inserted into a vein of the forearm into tubes containing potassium ethylene diamine tetra acetic acid (K2‐EDTA) at the following times for metabolite characterization, total radioactivity and parent drug analysis: Pre‐dose, 0.5, 1, 1.5, 2, 3, 4, 6, 8, 12, 24, 48, 72, 96, 120 and 144 h post‐dose. Urine samples were collected into pre‐weighed, uniquely labeled containers pre‐dose (before drug administration) and at the following intervals: 0–4, 4–8, 8–24, 24–48, 48–72, 72–96, 96–120 and 120–144 h post‐dose. Feces samples were collected quantitatively into uniquely labeled, polypropylene containers pre‐dose (before drug administration) and individually until 144 h post‐dose. Urine and feces samples were frozen at −20°C and blood samples at −70°C.

### 2.5 Measurement of total radioactivity in plasma, blood, feces, and urine by liquid scintillation counting (LSC)

Analysis of total radioactivity data in plasma, blood, feces, and urine was performed by Covance Laboratories Inc. All sample combustions were performed in a Model 307 Sample Oxidizer (Packard Instrument Company) and the resulting ^14^CO_2_ was trapped in a mixture of Perma Fluor™ (Perkin Elmer) and Carbo Sorb® (Perkin Elmer). Oxidation efficiency was evaluated on each day of sample combustion by analyzing a commercial radiolabeled standard both directly in a scintillation cocktail and by oxidation. Acceptance criteria were combustion recoveries of 95 to 105% relative to the direct analysis. EcoLite™(+) scintillation cocktail was used for samples analyzed directly. All samples were analyzed for radioactivity in Model 2900TR or 2910TR liquid scintillation counters (Packard Instrument Company) for at least 5 min or 100 000 counts. Each sample was homogenized or mixed before radioanalysis (unless the entire sample was used for analysis). All samples were analyzed in duplicate or triplicate, as applicable, if sample size allowed. If results from sample replicates (calculated as ^14^C disintegrations per minute (dpm)/g sample) differed by more than 10% from the mean value and sample aliquots had radioactivity greater than 200 dpm, the sample was re‐homogenized or remixed and reanalyzed (if the sample size permitted).

Scintillation counts per minute (cpm) data were automatically corrected for counting efficiency using the external standardization technique and an instrument‐stored quench curve generated from a series of sealed quenched standards.

Blood samples were mixed and duplicate aliquots of each (approximately 0.4 g) were combusted and analyzed by liquid scintillation couLSC. Plasma and urine samples were separately mixed and duplicate weighed aliquots of each (approximately 0.4 g for plasma and 0.2 g for urine) were analyzed directly by LSC. Urine volumes provided by the Covance clinical research unit were entered as a weight assuming a specific gravity of 1 g/mL. Feces samples were analyzed individually (samples were not combined by interval) and the weight of each individual sample was recorded. A weighed amount of methanol:water (20:80, v:v; approximately 2 to 3 times the sample weight, such that the homogenate weight:sample weight ratio was approximately 3 to 4) was added and the sample was mixed and homogenized using a probe type homogenizer. Triplicate‐weighed aliquots (approximately 0.2 g) were combusted and analyzed by LSC. The empty treatment vial from each subject was analyzed by adding a weighed amount of methanol to each vial and analyzing duplicate weighed aliquots (approximately 0.5 g) of each extract by LSC. Residual radioactivity recovered from each treatment vial was subtracted from the dose administered to the appropriate subject.

### 2.6 Sample preparation

To the thawed plasma samples (1.5 mL plasma/ time points) two volumes of ice‐cold acetonitrile:ethanol:acetic acid (90:10:0.1, v/v) were added, followed by vortex mixing (10 min) and sonication (10 min). After centrifugation, the supernatants (4.2 mL) were evaporated to near dryness under a gentle stream of nitrogen on a TurboVap LV (Zymark Corp.). The residues were reconstituted in 80 μL of methanol:water:acetic acid (50:50:0.1, v/v) and 45 μL were injected into the ultra‐performance liquid chromatography (UPLC) system with off‐line radioactivity detection.

A pool of equal percent volume of urine (0–48 h) was prepared for each subject. An aliquot (1.5 mL) of the pooled sample was concentrated ~7‐fold by reducing the volume under nitrogen and then combined with 50 μL methanol:water:acetic acid (50:50:0.1; v/v) followed by centrifugation at 3500 rpm (~1000 × g) for 10 min. The aliquot was then injected onto the UPLC system with off‐line radioactivity detection.

A pool of approximately equal percent by weight of feces (from 0–96 h or 0–120 h) was prepared for each subject. Feces samples were extracted two times with 2 equivalent volumes of acetonitrile: each time with 10 min of vortexing, 10 min of sonication, and centrifugation at 3500 rpm (~1000 × g) for 10 min. An aliquot of the combined supernatant was concentrated approximately 5‐fold by evaporating to dryness under nitrogen and reconstituting in methanol:water:acetic acid (50:50:0.1, v/v) 45 μL of the concentrated, reconstituted samples was injected onto the UPLC system with off‐line radioactivity detection.

### 2.7 Determination of metabolite profiles and identification of metabolites

Characterization and quantification of the metabolites of encorafenib in select human plasma and excreta samples were performed by the Department of DMPK (Drug Metabolism and Pharmacokinetics) at the Novartis Institutes for Biomedical Research (East Hanover, NJ, USA) using liquid chromatography (LC) coupled to radioactivity detectors and mass spectrometers (MS).

The analytical system consisted of a Waters ACQUITY UPLC system (Waters), equipped with a Phenomenex Luna PFP column (3 μm, 4.6 × 150 mm) (Phenomenex) and a guard column of the same phase, both maintained at a temperature of 30°C. The mobile phases consisted of 10 mM ammonium acetate containing 0.1% formic acid (v/v) (solvent A) and CH_3_CN (solvent B). The total run time was 80 min. A linear gradient elution program was used with a flow rate of 1 mL/min as described in Table 1.

**TABLE 1:** HPLC gradient for [^14^C] encorafenib radio‐chromatography.

Time (minutes)	Solvent A (%)	Solvent B (%)
Initial	97	3
4	97	3
10	85	15
55	62	38
65	5	95
70	5	95
70.5	97	3
80	97	3

The UPLC effluent was split 4:1, with 200 μL of the total flow introduced to the mass spectrometer for mass spectral analysis and the remaining flow used for radioactivity detection.

For off‐line radioactivity detection, the eluant was collected into 96‐well Lumaplate (Packard Instrument Co) microplates using a Collect Pal fraction collector (Leap Technologies) with a collection time of 10 seconds per well. The LumaPlates were sealed after drying and counted for 10–50 min per well on a Packard Topcount microplate scintillation counter. The structural characterization of metabolites was carried out using the above UPLC profiling method coupled to a two‐channel Z‐spray (LockSprayTM) time‐of‐flight mass spectrometer (Synapt MS). Leucine enkephalin was used as the mass reference standard for exact mass measurements and was delivered via the second spray channel at a flow rate of 5 μL/minute. The mass spectrometer was operated at a resolution of ~10 000 m/ΔmFWHM with spectra being collected from 100 to 1000 amu. The ionization technique employed was positive electrospray. The sprayer voltage was kept at 3100 V and the sampling cone voltage was kept at a potential of 20 V. For the time‐of‐flight MS/MS experiments, trap energies of 25 to 45 eV and transfer energy of 15 eV were used with argon as the collision gas.

All radioactivity data were transferred to Laura software (version 4.2.2.24, Lab Logic Ltd, UK) to generate and integrate radio‐chromatograms. Radioactive peaks for quantitative analysis were manually selected from the plots. The radioactivity in the region encompassing the beginning and end of the peak was summed. All further calculations were based on radioactivity.

The percent of radioactivity (%RA) in a particular peak, *Z*, was calculated as follows in Equation (1):

(1) $$\%\mathrm{RA}\;\text{in}\;Z=\frac{\mathrm{DPM}\;\text{in Peak}\;Z}{\text{total}\;\mathrm{DPM}\;\text{in}\;\mathrm{all}\;\text{integrated peaks}}x\;100$$

The concentration or amount of each component was calculated as % RA (expressed as a fraction) multiplied by the total concentration of radioactivity in plasma, or the percent of dose in the excreta.

### 2.8 Pharmacokinetic data analysis

The total radioactivity in blood and plasma was determined by LSC. Nominal sampling time points were used for deriving total radioactivity blood and plasma PK data. PK parameters (maximum concentration (*C*_max_), time to maximum concentration (*T*_max_), terminal half‐life(*t*_1/2_) time to last measurable concentration (*T*_last_), area under the curve to the last measurable time point (AUC_last_), area under the curve extrapolated to infinity (AUC_inf_)) describing blood and plasma total radioactivity were determined using non‐compartmental methods via WinNonlin® Professional (Version 6.3, Pharsight). Descriptive statistics of PK parameters included mean, median, standard deviation (SD), percent coefficient of variation (CV%) and range. Missing values of concentrations were not imputed and concentrations below the lower limit of quantitation (LLOQ) were treated as zero. When summarizing concentrations, zero values were excluded from the calculation of geometric means and geometric CVs. However, they were included in all other summary statistics and the number of non‐zero concentrations was reported. Missing values for any PK parameters were not imputed and were handled as missing values.

### 2.9 Stability in simulated gastric and intestinal fluid

Simulated gastric fluid (SGF) was generated by mixing 2.0 g of sodium chloride and 3.2 g of purified pepsin, which is derived from porcine stomach mucosa, with an activity of 800 to 2500 units per mg of protein, in 7.0 mL of hydrochloric acid and sufficient water to make 1000 mL. The final test solution has a pH of about 1.2. Simulated intestinal fluid (SIF) was generated by dissolving 6.8 g of monobasic potassium phosphate in 250 mL of water and adding 77 mL of 0.2 N sodium hydroxide and 500 mL of water. To this mixture, 10.0 g of pancreatin was added and the solution pH was adjusted with either 0.2 N sodium hydroxide or 0.2 N hydrochloric acid to a pH of 6.8 ± 0.1 and diluted with water to a final volume of 1000 mL. Encorafenib was dissolved in SGF (100 μM final encorafenib concentration) and incubated for 1 h or SIF (1 μM final encorafenib concentration) for 3 h. Dilutions are prepared in HPLC vials by combining 20 μL of the test compound in DMSO with 180 μL of the specified assay media. Separate dilutions are prepared for each time point per media. The media is mixed by repeated pipetting, vortexed and incubated at 37°C. At the specified time period and equal volume of cold ACN is added to the vial to precipitate the protein and quench any enzymatic activity. The vials are spun and an aliquot of supernatant is removed and plated in a 96‐well plate for analysis. An Agilent 1100 HPLC (Agilent, Santa Clara, CA, USA) equipped with a micro‐well plate autosampler, quaternary HPLC pump, and diode array detector was used for analysis. Each supernatant (60 μL) was injected onto the column (AQUASIL C18, 3 μM 50 × 2.1 mm (Fischer Scientific)) and eluted using water with 0.1% trifluoroacetic acid (solvent A) and acetonitrile with 0.1% trifluoroacetic acid (solvent B) the gradient program in Table 2 below. Data were collected at 214, 254, 280 and 320 nm. Results are reported using data obtained at 320 nm. Stability is calculated as % remaining relative to the T = 0 peak area.

**TABLE 2:** Retention times obtained by HPLC analysis of solvent mixture.

Time (minutes)	Solvent A (%)	Solvent B (%)
0	97	3
1.5	97	3
2.5	85	15
4.0	62	38
4.1	5	95
5.5	5	95

*Note*: Data was collected at 214, 230, 254, and 280 nm.

## 3 RESULTS

### 3.1 Subject demographics and disposition

A total of four subjects were enrolled in the study. All four (100%) subjects completed the study per protocol. The mean age of the subjects was 33.8 years (range: 32 to 35 years). All subjects were male; three Caucasians (75.0%) and one Asian (25.0%). The mean body mass index (BMI) for enrolled patients was 25.3 (range: 23.7 to 26.3 kg/m^2^).

Overall, three subjects (75.0%) experienced at least one adverse event (AE) during the study. No grade 2, 3 or 4 AEs were reported. Two subjects (50.0%) experienced AEs suspected to be related to the study drug. The reported AEs suspected by the investigator to be related to the study drug included flushing, facial edema and erythema, peripheral edema, palmar erythema, keratosis pilaris, and erythematous rash.

### 3.2 Mass balance

**FIGURE 2:**
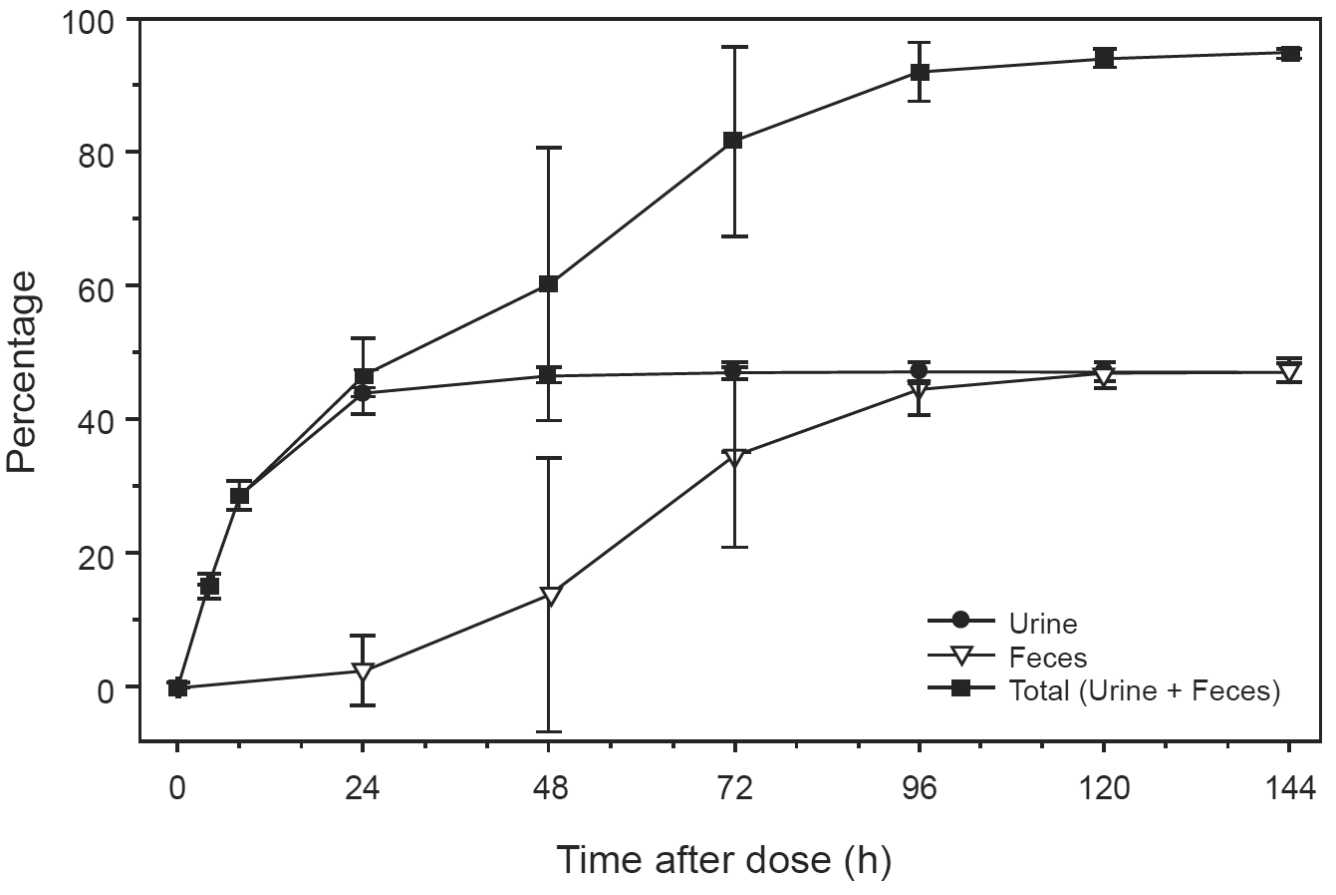
Mean cumulative percent of radioactive dose from all four subjects recovered in urine and feces following administration of a single dose of 100 mg of [^14^C] encorafenib to healthy male subjects in the pharmacokinetic analysis set (linear scale). Error bars are presented as ± SD and have been staggered.

The mean cumulative percent excretion of radioactivity from all four subjects is presented in Figure 2. Overall, good mass balance was achieved in all four subjects (94.5 ± 0.63% dose) by the end of Day 6 (144 h post‐dose). An equal mean of 47.2% of the dose was recovered in urine and feces through the last collection interval.

### 3.3 Radiolabeled plasma/blood PK

**FIGURE 3:**
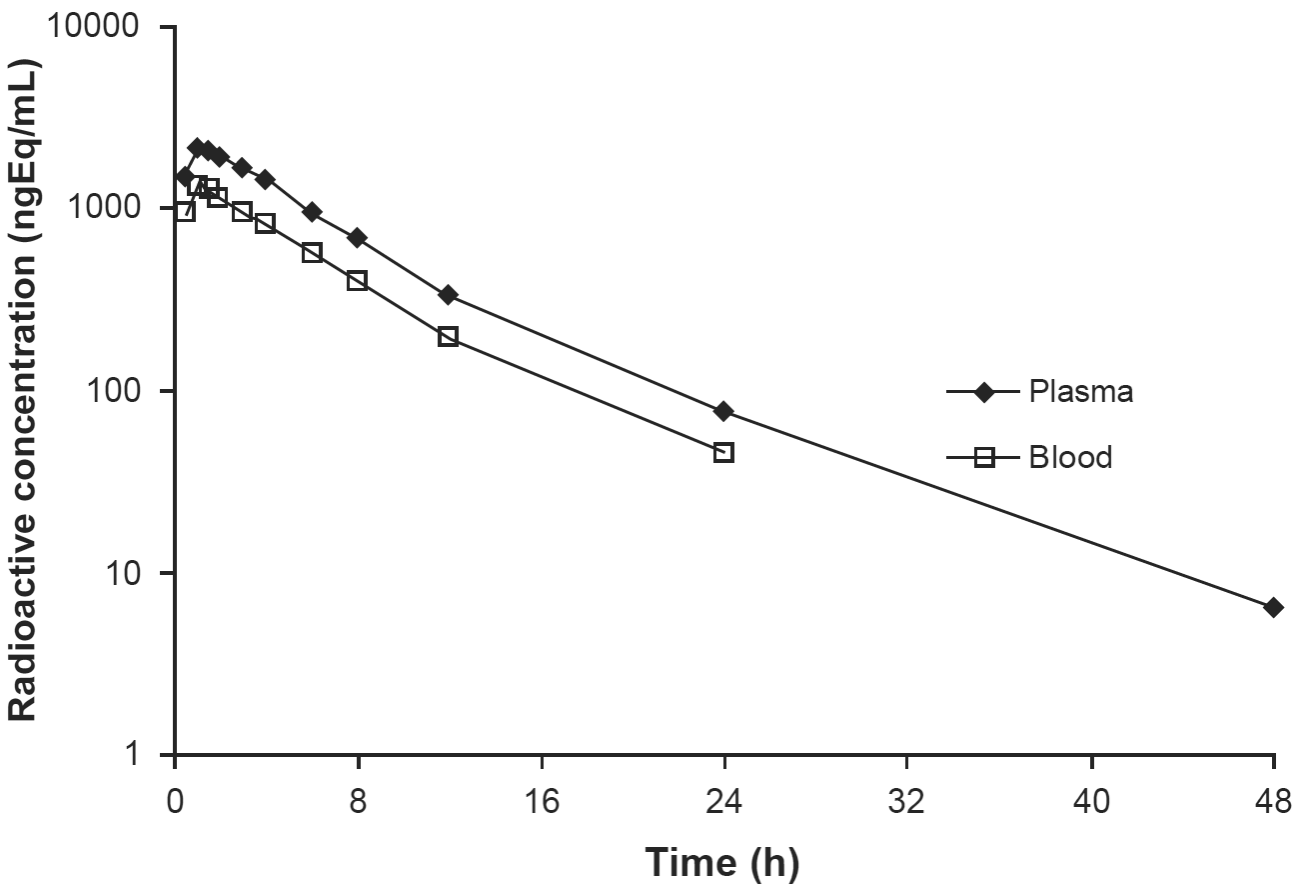
Plasma and whole blood concentration‐versus‐time profiles for a single oral dose of 100 mg of radiolabeled single oral dose of 100 mg (90 μCi) of [^14^C] encorafenib.

Concentrations of total radioactivity in blood and plasma were measured by LSC at the clinical site. The mean total radioactivity concentration‐time data in blood and plasma is presented in Figure 3 alongside the PKs of non‐radiolabeled encorafenib (described above). Following a single oral dose of 100 mg of [^14^C] encorafenib, the median total radioactivity peak concentration was reached (T_max_) at a median time of 1.0 h (range: 1.0–1.5 h) in blood and a median time of 1.3 h (range: 1.0–1.5 h) in plasma (Table 3). The mean terminal half‐lives of radioactivity in blood and plasma were similar, 4.8 h and 5.1 h, respectively. Summary statistics of non‐radiolabeled encorafenib PK parameters are presented in Table 4. The mean [blood]:[plasma] AUC ratio for total radioactivity was 0.58, suggesting that drug‐related radioactivity has a preferential distribution to plasma when compared with blood. This value was also in line with the [blood]/[plasma] value of 0.75 ± 0.21 determined in an in vitro study (data on file). Similar values of mean (SD) [blood]/[plasma] were obtained from 1 to 24 h post‐dose in the current study (0.59 [0.02]).

**TABLE 3:** Summary statistics of plasma and whole blood PK parameters for total radioactivity.

	Plasma				Whole Blood			
Statistic	AUC_inf_ (ngEq*h/mL)	*C*_max_ (ngEq/mL)	*T*_max_ (h)	*T*_1/2_ (h)	AUC_inf_ (ngEq*h/mL)	*C*_max_ (ngEq/mL)	*T*_max_ (h)	*T*_1/2_ (h)
*n*	4	4	4	4	4	4	4	4
Mean (SD)	15 600 (3780)	2170 (107)	NR	5.1 (1.30)	8950 (1310)	1330 (103)	NR	4.8 (0.48)
Median (Minimum–Maximum)	14 300 (12 800–21 200)	2190 (2020–2330)	1.0 (1.0–1.5)	4.5 (7.1–4.3)	8760 (7670–10 600)	1320 (1230–1460)	1.0 (1.0–1.5)	4.6 (4.5–5.5)

**TABLE 4:** Summary statistics of plasma PK parameters for non‐radiolabeled encorafenib.

Statistics	AUC_inf_ (ng*h/mL)	AUC_last_ (ng*h/mL)	AUC_0‐24_ (ng*h/mL)	*C*_max_ (ng/mL)	*T*_max_ (h)	*T*_1/2_ (h)	CL/*F* (L/h)	Vz/*F* (L)
*n*	4	4	4	4	4	4	4	4
Mean (SD)	3490 (1430)	3910 (1450)	3850 (1370)	1050 (171)	NR	6.11 (1.84)	27.9 (9.15)	235 (73.7)
GeoMean (GeoCV%)	3750 (36.3)	3720 (37.1)	3680 (35.7)	1040 (17.3)	NR	5.88 (34.0)	26.6 (36.6)	226 (32.7)
Median (Minimum–Maximum)	3610 (2660–5860)	3600 (2600–5840)	3570 (2600–5670)	1080 (820–1230)	0.767 (0.500–1.00)	6.32 (3.74–8.09)	28.4 (17.1–37.6)	228 (168–316)

### 3.4 Non‐radiolabeled plasma PK

**FIGURE 4:**
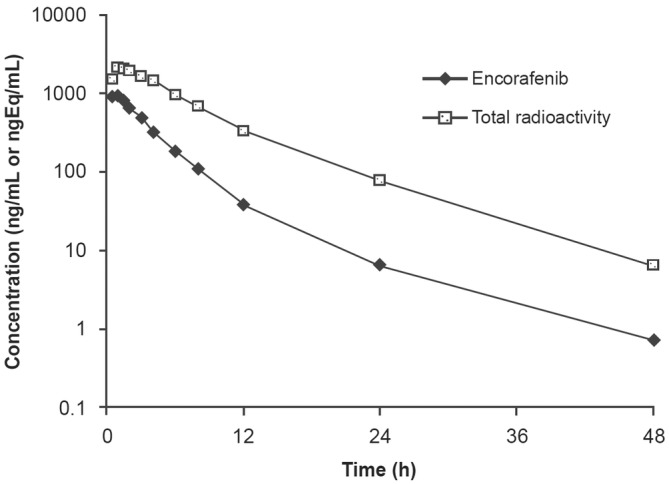
Plasma concentration‐versus‐time profiles of mean total radioactivity and non‐radiolabeled encorafenib for a single oral dose of 100 mg (90 μCi) of [^14^C] encorafenib.

Plasma concentration‐time curves for a single oral dose of 100 mg of non‐radiolabeled encorafenib are presented in Figure 4, alongside total radioactivity (see below). Encorafenib was rapidly absorbed with a median time to maximum concentration (*T*_max_) of 0.77 h (range: 0.5–1.0 h). Terminal disposition of [^14^C] encorafenib indicated a mean half‐life of 6.11 h (range: 3.74–8.09 h). The geometric mean (range) of clearance (CL/*F*) and volume (Vz/*F*) was approximately 26.6 L/h (17.1–37.6 L/h) and 226 L (168–316 L), respectively. Summary statistics of non‐radiolabeled encorafenib PK parameters are presented in Table 4.

### 3.5 Metabolite profiles in plasma

**FIGURE 5:**
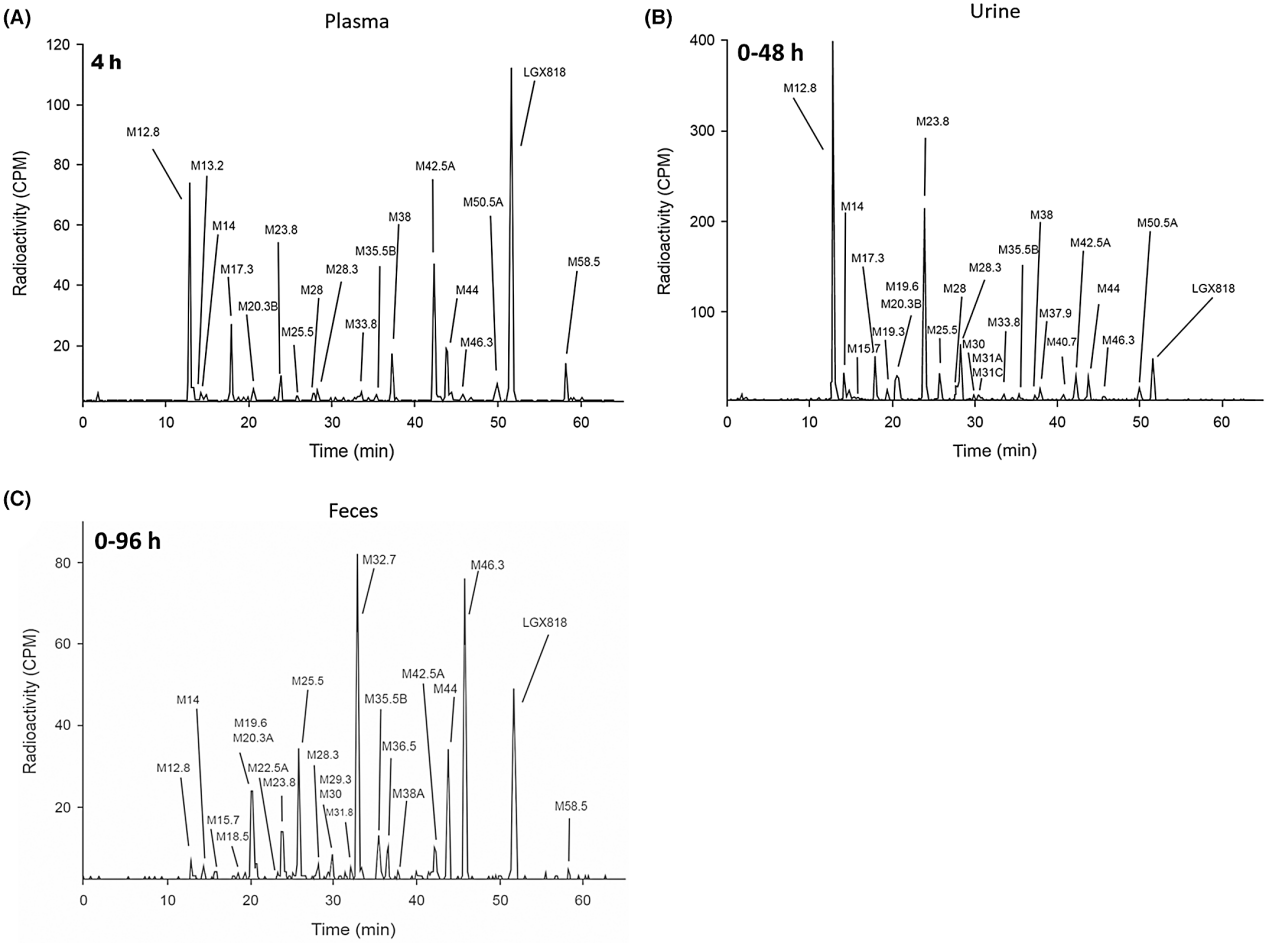
Representative radio‐chromatographic traces of metabolite profile in (A) plasma at 4 h post‐dose and (B, C) in pooled excreta, (B) urine (0–48 h), and (C) feces (0–96 h) following administration of a single dose of 100 mg (90 μCi) [^14^C] encorafenib to healthy male subjects.

After the oral administration of encorafenib to humans, encorafenib and approximately 20 metabolites were identified in plasma. The complexity of encorafenib plasma profiles was partly due to the multiple biotransformation pathways that occurred. These included: monohydroxylation, N‐dealkylation (loss of the isopropyl moiety and/or loss of the isopropylcarbamic acid methyl ester side chain), indirect glucuronidation, carbamate hydrolysis, oxidative deamination, reduction and dehydrogenation. Metabolites formed from combinations of these various biotransformation processes were also observed. A representative radio‐chromatographic trace of metabolite profile in plasma at 4 h post‐dose is shown in Figure 5A.

**FIGURE 6:**
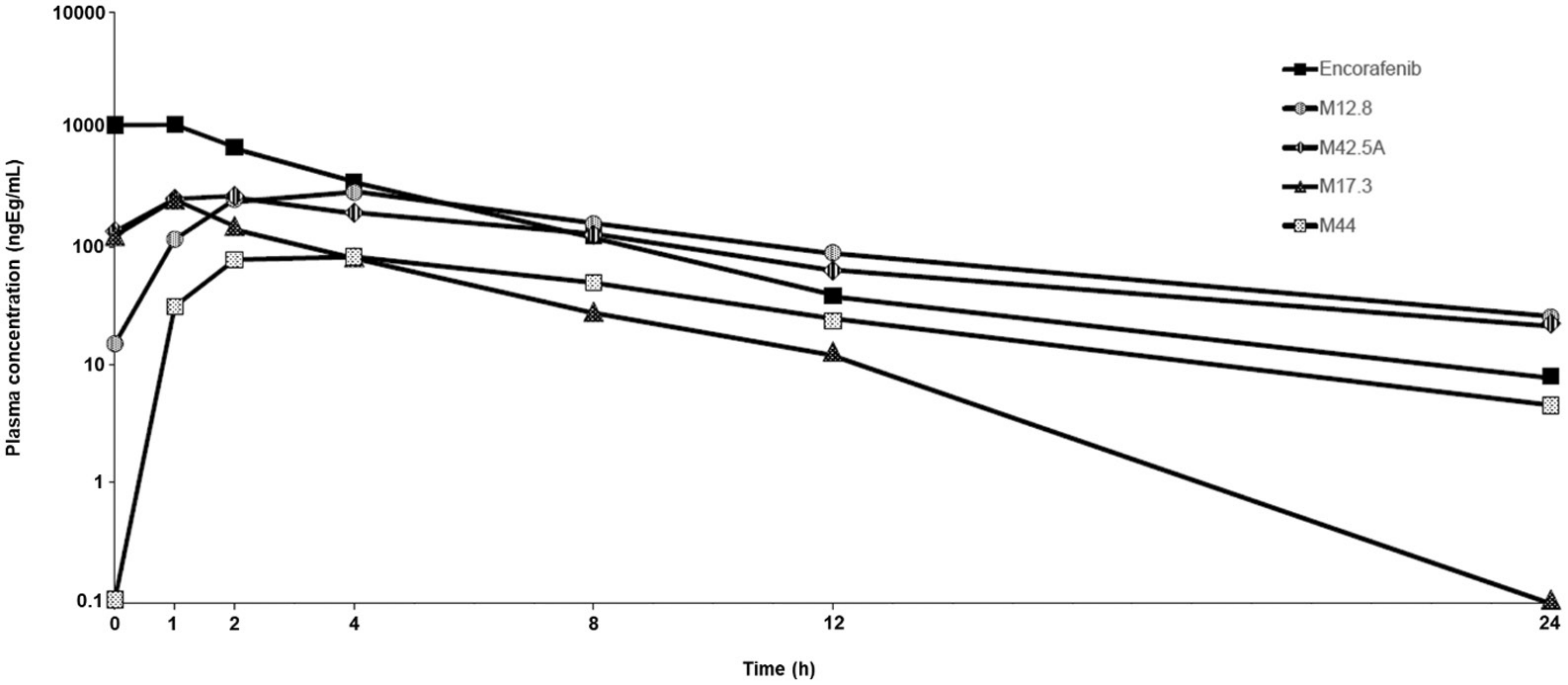
Plasma‐concentration time profiles of encorafenib and its major metabolites (M12.8, M42.5A, M17.3, M44) in plasma at 0, 1, 2, 4, 8, 12, and 24 h.

The most abundant radiolabeled component was encorafenib, with an average circulating radioactivity in plasma based on area under the curve from time zero to 24 h post‐dose (AUC_0‐24_) value of 27.5% (range: 17.9%–36.4%) (Table 5). The major circulating metabolites were M12.8, M42.5A, M17, and M44 (Table 5) and are shown in concentration‐time profiles with encorafenib (Figure 6). All other circulating metabolites were present at lower levels, with no individual metabolite contributing >4% to the radioactivity AUC (mean values). The AUC values reported for M12.8 were the sum AUC of M12.8 and M13.2, with M12.8 and M13.2 accounting for 19.3% and 3.68% of the total radioactivity AUC, respectively (Table 5). In addition, a small amount of the uncharacterized metabolite (M2) was detected, accounting for 0.76% of the AUC_0‐24h_ values. The structure of metabolite M2 could not be determined due to its low abundance in the sample. M2 could likely be the methane sulfonic acid formed from oxidative desulfonylation of encorafenib, due to the observation of metabolite M49 at trace levels (loss of the hydrosulfonyl methane radiolabel moiety) in plasma and excreta.

**TABLE 5:** Metabolite identification of major metabolites and relative AUC_0‐24_ in plasma.

Drug/Metabolite ID	Mean (range) Circulating Radioactivity in Plasma Based on AUC_0‐24_ (ngEq/mL*h)	Mean (range) Circulating Radioactivity in Plasma Based on AUC_0‐24_ (%) (range)	Notes
Encorafenib (LGX818)	4400 (2340–7140)	27.5 (17.9–36.4)	Parent compound
M12.8	2880 (2610–3290)	23.0 (16–29.3)	Indirect glucuronide of encorafenib
M42.5A	2430 (1740–3320)	15.5 (13.4–17.4)	LHY746; derived from the loss of the isopropyl carbamic acid methyl ester
M17.3	947 (818–1050)	6.22 (5.34–7.05)	Indirect glucuronide of encorafenib
M44	806 (525–1250)	5.09 (4.02–6.36)	Derived from carbamate hydrolysis followed by deamination and reduction
Total radioactivity	15 500 (13 100–19 600)	100 (NA)	

### 3.6 Metabolite profiles in excreta

In the four subjects from this study, an equal mean of 47.2% of the administered radioactive dose was recovered in the urine and feces through the last collection interval. After the oral administration of encorafenib to humans, there were at least 30 radiolabeled metabolites of encorafenib characterized and quantified in the excreta. Representative traces of pooled excreta including urine (0–48 h) and feces (0–96 h) are shown in Figure 5B,C, respectively.

In the urine, encorafenib ranged from 0.9 to 2.5% of the administered radioactive dose, with an average value of 1.8%. Renal clearance (CLr) of encorafenib was therefore judged to be minimal. The mean CLr for encorafenib was estimated by dividing the mean amount of encorafenib excreted in the urine (1.8 × 106 ng) by the mean plasma AUC_inf_ of encorafenib (3940 ng*h/mL). The estimated mean CLr of encorafenib (0.5 L/h) was only 7% of the glomerular filtration rate (7.5 L/h), suggesting that there was minimal involvement of renal transporters in the elimination process of encorafenib. The most abundant metabolites detected in the urine were M12.8 (indirect glucuronide of encorafenib, ~12 to ~15% of dose) and M23.8 (a double N‐dealkylated metabolite derived from the loss of both the isopropyl moiety and the isopropyl‐carbamic acid methyl ester side chain, ~11 to ~14% of dose). All other metabolites were present at ≤3.2% of the dose.

**FIGURE 7:**
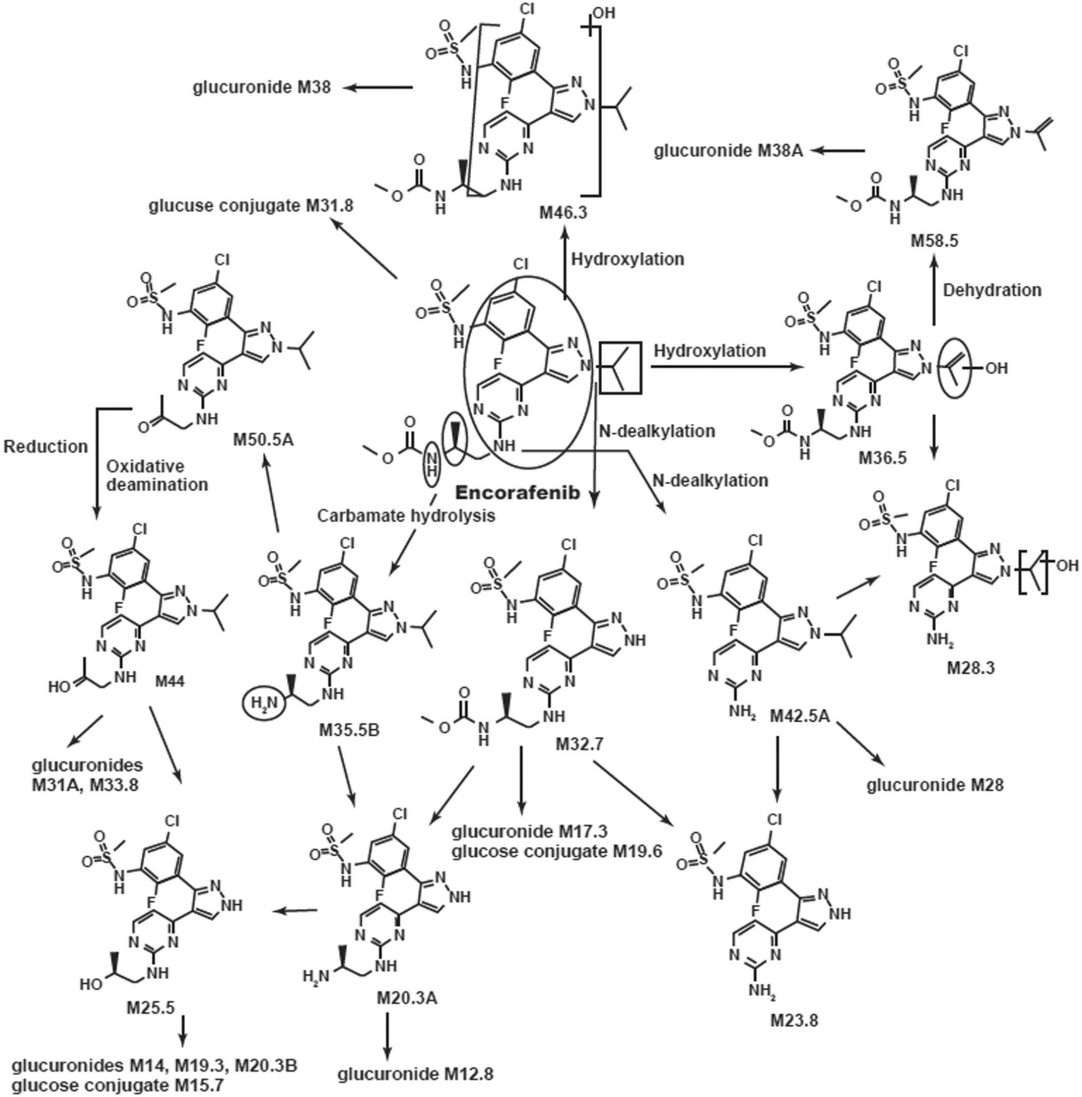
Biotransformation pathways for encorafenib (LGX818).

In the feces, encorafenib accounted for 4.2% to 6.1% of the administered radioactive dose, with an average value of 5.0%. The fecal profiles were also complex showing M19.6 (glucose conjugate of M32.7), M25.5 (N‐dealkylation and carbamate hydrolysis followed by oxidative deamination and reduction), M32.7 (LII306; N‐desisopropyl encorafenib), M44 and M46.3 (monohydroxy‐encorafenib) as the most prominent metabolites, accounting for ~3.2 to ~7.2% of the dose (M19.6), ~3.0 to ~5.2% of the dose (M25.5), ~8.0 to ~12% of the dose (M32.7), ~2.6 to ~3.3% of the dose (M44), and ~ 3.4 to ~6.9% of the dose (M46.3). All other metabolites were present at ≤1.8% of the dose. Refer to Figure 7 for the proposed biotransformation scheme of encorafenib in humans.

### 3.7 Stability in SIF and SGF

Following incubation in SIF and SGF‐containing enzymes at encorafenib concentrations reasonably expected to be observed in the stomach and the intestine during the absorption phase of administration, encorafenib appeared to be stable. The approximate percentage of encorafenib remaining following a 3‐h incubation in SIF with pancreatin was approximately 97% relative to the initial incubation (time = 0 h). Similar results were observed for the SGF with pepsin, with approximately 101% encorafenib remaining relative to the initial incubation (time = 0 h).

## 4 DISCUSSION

The present study was an open‐label, single‐center Phase I study to determine ADME properties of encorafenib following a single oral administration of 100 mg encorafenib containing 90 μCi of [^14^C] in four healthy male subjects. A single‐dose administration was shown to be safe and well‐tolerated in healthy male subjects. No clinically relevant AEs were reported during the study. There were no significant abnormalities in laboratory evaluations, vital signs or electrocardiograms (ECG).

Following a single oral dose of 100 mg of encorafenib, the overall recovery of radioactivity in the excreta was high (> 90%) in all 4 subjects, indicating that good mass balance was achieved. Lack of complete recovery may be due to residual material remaining in the body, as evaluation of excreted radioactivity was limited to 144 h in this study. This would be consistent with detectible levels of radioactivity in the liver of rats in a quantitative whole‐body autoradiography study. An equal mean of the radioactivity dose was eliminated in the feces and the urine. The percentage of the dose eliminated in the urine as unchanged encorafenib was minor, thus CLr of encorafenib was judged to be minimal. The mean percentage of the dose eliminated in the feces as unchanged encorafenib was also minor. Thus, metabolism was found to be the major clearance pathway (~88% of the recovered radioactive dose) for encorafenib in humans. Additionally, it is reasonable to anticipate that metabolites found in feces may also be a result of gut extraction of encorafenib by intestinal CYP enzymes.

Encorafenib was primarily metabolized by CYP3A4 (83%) as the main contributor, with minor contributions from CYP2C19 (16%) and CYP2D6 (1%).^10^ The major circulating metabolites above with exposures of approximately 10% of total circulating radioactivity were M12.8, an indirect glucuronide of encorafenib of minimal pharmacologic concern, and the primary phase 1 metabolite (M42.5A, also known as LHY746), formed primarily by CYP3A4 (data on file). Based on single‐dose clinical and non‐clinical studies with [^14^C] encorafenib and the assumption of linear PK, similar exposures at the 450 mg QD dose in humans and no observed adverse effect level (NOAEL) dose levels in the rat and monkey long‐term toxicology evaluations were observed (data on file). Secondary pharmacology results indicate that M42.5A (also known as LHY746) is approximately 1/30th of the potency of encorafenib against A375 (BRAF V600E) melanoma cell proliferation when compared with encorafenib and that it shows no significant safety signal in in vitro safety pharmacology testing (data on file). Steady‐state exposures of M42.5A (LHY746) are being assessed in a clinical study (NCT03864042).^11^

The oral absorption was estimated from the radioactive dose recovered in the urine and the total radioactive dose recovered in the feces as metabolites (mostly oxidative metabolites and their Phase II conjugates). The mean radioactivity dose recovered in the urine was 47%, and the mean radioactivity dose recovered in the feces in the form of metabolites was 39%. Based on these values and the assumptions that encorafenib and its metabolites are stable in feces and the gastrointestinal tract, as supported but the in vitro SGF/SIF assay which indicated no appreciable degradation of the compound, the fraction of oral absorption (Fa) was estimated to be at least ~86%.

The observed PK parameters in healthy subjects in this study were slightly lower, at the same 100‐mg dose than the values presented prior in patients with cancer based on a comparison of the median (range) across studies.^8^ Additionally, higher variability was observed for the apparent oral clearance presented in the encorafenib labeling (54%) relative to the value reported in this study (37%).^10^ These differences may be a result of a few key differences between this phase 1 study and the broader oncology development program. Of note, the use of a microemulsion liquid oral suspension in this study versus a filled, formulated capsule in broader development in oncology patients may play a role. It is also notable that the differences in underlying health status, including allowing broader inclusion of cancer patients with elevated baseline liver function values in the dose escalation oncology study relative to those healthy subjects enrolled in this study, could explain this difference, particularly for a drug with a substantial metabolic contribution to elimination. Finally, the limited number of subjects in this study may also impact these comparisons.

An additional design characteristic of this study was the selection of the dose of encorafenib (100 mg). The dose used in this healthy subjects study was approximately 3‐fold lower than the dose approved (300 mg) for the treatment of metastatic colorectal cancer and 4.5‐fold lower than the dose approved (450 mg) for the treatment of metastatic melanoma.^10^ At the time the study was conducted, the highest tested dose in the microemulsion formulation was 100 mg. Although higher doses would more closely mimic the approved clinical doses of encorafenib, intake of higher doses of cremophor, the emulsifying agent, was known to be linked with an increased risk of gastrointestinal discomfort, thereby limiting the total daily dose of encorafenib that can be given as a microemulsion. After a single dose, systemic exposure of encorafenib was dose‐proportional over the dose range of 50 mg to 700 mg, further suggesting that results may be reasonably extrapolated from the tested dose (100 mg) to the clinically relevant doses (300–450 mg).^10^ Moreover, the practice of using a lower dose in the radiolabel ADME study, relative to a patient study, is not entirely uncommon in oncology. A recent review of late development stage/approved oncology drugs, including many targeted therapies, found 10 ADME studies of the 40 evaluated (25%) were conducted at a dose that was considered lower than the therapeutic dose.^12^

In summary, encorafenib was rapidly absorbed and had a reasonable elimination time to support once daily dosing. Encorafenib was primarily eliminated via metabolism of the parent compound, suggesting a key role in hepatic elimination.

## AUTHOR CONTRIBUTIONS

Participated in research design. Performed data analysis: Lance Wollenberg, Erik Hahn, Jason Williams, Kevin Litwiler. Wrote or contributed to the writing of the manuscript: Lance Wollenberg, Erik Hahn, Jason Williams, Kevin Litwiler.

## DATA AVAILIBILITY STATEMENT

Upon request, and subject to review, Pfizer will provide the data supporting the findings of this study. Subject to certain criteria, conditions and exceptions, Pfizer may also provide access to the related individual de‐identified participant data. See https://www.pfizer.com/science/clinical‐trials/trial‐data‐and‐results for more information.

## DISCLOSURE STATEMENT

All authors are employees of Pfizer, Inc. and may own Pfizer stock.

## References

[1] Davies H , Bignell GR , Cox C , et al. Mutations of the BRAF gene in human cancer. Nature. 2002;417:949‐954. 12068308 10.1038/nature00766

[2] Flaherty KT , Puzanov I , Kim KB , et al. Inhibition of mutated, activated BRAF in metastatic melanoma. N Engl J Med. 2010;363:809‐819. 20818844 10.1056/NEJMoa1002011 PMC3724529

[3] Melo M , Gaspar da Rocha A , Batista R , et al. TERT, BRAF, and NRAS in primary thyroid cancer and metastatic disease. J Clin Endocrinol Metab. 2017;102:1898‐1907. 28323937 10.1210/jc.2016-2785

[4] Myall NJ , Henry S , Wood D , et al. Natural disease history, outcomes, and Co‐mutations in a series of patients with BRAF‐mutated non‐small‐cell lung cancer. Clin Lung Cancer. 2019;20:e208‐e217. 30442523 10.1016/j.cllc.2018.10.003 PMC6387831

[5] Sanz‐Garcia E , Argiles G , Elez E , Tabernero J . BRAF mutant colorectal cancer: prognosis, treatment, and new perspectives. Ann Oncol. 2017;28:2648‐2657. 29045527 10.1093/annonc/mdx401

[6] Hauschild A , Grob JJ , Demidov LV , et al. Dabrafenib in BRAF‐mutated metastatic melanoma: a multicentre, open‐label, phase 3 randomised controlled trial. Lancet. 2012;380:358‐365. 22735384 10.1016/S0140-6736(12)60868-X

[7] Kopetz S , Grothey A , Yaeger R , et al. Encorafenib, binimetinib, and cetuximab in BRAF V600E‐mutated colorectal cancer. N Engl J Med. 2019;381:1632‐1643. 31566309 10.1056/NEJMoa1908075

[8] Delord JP , Robert C , Nyakas M , et al. Phase I dose‐escalation and ‐expansion study of the BRAF inhibitor encorafenib (LGX818) in metastatic BRAF‐mutant melanoma. Clin Cancer Res. 2017;23:5339‐5348. 28611198 10.1158/1078-0432.CCR-16-2923

[9] Sullivan RJ , Weber J , Patel S , et al. A phase Ib/II study of the BRAF inhibitor encorafenib plus the MEK inhibitor binimetinib in patients with BRAF(V600E/K) ‐mutant solid tumors. Clin Cancer Res. 2020;26:5102‐5112. 32669376 10.1158/1078-0432.CCR-19-3550

[10] Lemery SJ . BRAFTOVI® (Encorafenib, [Package Insert]). Array BioPharma Inc; 2020.

[11] Pfizer . Pharmacokinetic Drug‐drug Interaction Study of Encorafenib and Binimetinib on Probe Drugs in Patients With BRAF V600‐mutant Melanoma or Other Advanced Solid Tumors. ClinicalTrials.gov identifier: NCT03864042. Updated November 25, 2022. Accessed December 05, 2022. https://clinicaltrials.gov/ct2/show/NCT03864042

[12] Nijenhuis CM , Schellens JH , Beijnen JH . Regulatory aspects of human radiolabeled mass balance studies in oncology: concise review. Drug Metab Rev. 2016;48:266‐280. 27186889 10.1080/03602532.2016.1181081

